# Association Between Self‐Reported Sleep Quality and Conversion From Mild Cognitive Impairment to Dementia: A Retrospective Cohort Study Using the English Longitudinal Study of Ageing

**DOI:** 10.1002/brb3.71120

**Published:** 2025-12-17

**Authors:** Johannes Schurr, Hilary Davies‐Kershaw, Helen Strongman

**Affiliations:** ^1^ London School of Hygiene & Tropical Medicine London UK; ^2^ Department of Medicine I LMU University Hospital, Ludwig‐Maximilians‐Universität Munich Germany; ^3^ Department of Population Health London School of Hygiene & Tropical Medicine London UK; ^4^ Department of Non‐Communicable Disease Epidemiology London School of Hygiene & Tropical Medicine London UK

**Keywords:** dementia, mild cognitive impairment, older adults, sleep duration, sleep quality

## Abstract

**Purpose:**

Poor sleep quality is common in people diagnosed with mild cognitive impairment (MCI) and dementia and may be associated with conversion to dementia.

**Method:**

This retrospective cohort study investigated the association of self‐reported sleep quality and duration with dementia incidence in a cohort of people aged ≥ 50 with MCI from the English Longitudinal Study of Ageing (ELSA) panel study. We identified the MCI cohort using three waves (2008/2009, 2012/2013, 2016/2017) based on absence of diagnosed dementia, self‐reported memory problems, preserved ability for daily activities, and reduced cognitive function in neuropsychological assessments. Exposures were self‐reported poor sleep quality and sleep duration in the month before the baseline interview. The outcome was self‐reported physician‐diagnosed dementia from later waves. We modeled associations with Cox proportional hazards regression adjusted for multiple confounders.

**Finding:**

Among 1885 patients with MCI at baseline, 24.7% reported poor sleep quality, 17.6% reported average sleep duration per night to be < 6 h, 74.6% reported average sleep duration per night to be 6–< 9 h, and 7.5% reported average sleep duration per night to be ≥ 9 h. Note that 176 (9.3%) developed dementia during follow‐up (median 5.8 years, interquartile range [IQR] 2.1–6.3). Age‐adjusted hazard for all‐cause dementia, compared to 6–< 9 h sleep duration, was increased with sleep duration ≥ 9 h (hazard ratio [HR] 2.57, 95% confidence interval [CI] 1.62–4.02) but not short sleep duration (HR 0.94, 95% CI 0.55–1.61); there was weak evidence of reduced risk in persons with poor sleep quality, compared to good sleep quality (HR 0.68, 95% CI 0.41–1.12).

**Conclusion:**

We found evidence for an association between long sleep duration and dementia risk in patients with MCI and limited evidence of an association between poor sleep quality and dementia.

## Graphical abstract

This study examined the association between self‐reported sleep quality, sleep duration and the incidence of dementia in adults aged 50 and older with mild cognitive impairment (MCI), using data from the English Longitudinal Study of Ageing. Results showed that longer sleep duration (≥ 9 h per night) was associated with a higher incidence risk of all‐cause dementia, whereas short sleep duration (< 6 h) was not strongly linked to increased dementia incidence. There was weak evidence suggesting a reduced risk of dementia among individuals reporting poor sleep quality. Overall, the findings indicate that long sleep duration may increase dementia risk among people with MCI, while evidence for an association between poor sleep quality and dementia remains limited.

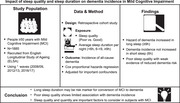

**Abbreviations**
AD: Alzheimer's disease
ADNI: Alzheimer's Disease Neuroimaging Initiative
CES‐D: Center for Epidemiologic Studies Depression Scale
CI: confidence interval
CVD: cardiovascular disease
ELSA: English Longitudinal Study of Ageing
HR: hazard ratio
IQCODE: Informant Questionnaire on Cognitive Decline in the Elderly
IQR: interquartile range
IR: incidence rate
MCI: mild cognitive impairment
NACC: National Alzheimer's Coordinating Center
py: person‐year

## 1 Introduction

Mild cognitive impairment (MCI) is a disorder involving mild but noticeable memory problems that are beyond those expected at the individual’ s age (Winblad et al. 2004). It is characterized by a cognitive decline but preserved functional abilities to perform activities of daily life (Albert et al. 2011; Petersen et al. 2014). People diagnosed with MCI have a higher risk for developing dementia, compared to people with normal cognition, and show an annual conversion rate of approximately 5% in community settings (Bermejo‐Pareja et al. 2020). Nonetheless, some remain in a stable stage or even show an improvement of their cognitive status over time (Bermejo‐Pareja et al. 2020). Sleep disturbances, expressed as changed sleep patterns resulting in decreased sleep efficiency, are frequently correlated with MCI (Brachem et al. 2020; Choe et al. 2022) and dementia syndromes, including Alzheimer's disease (AD) (Yaffe et al. 2014; Bubu et al. 2017; Xu et al. 2020).

It has been hypothesized that poor sleep quality might be an early predictor not only for the transition from normal cognition to MCI, but also from MCI to dementia (Romanella et al. 2021; Casagrande et al. 2022). Cohort studies analyzing the association between sleep problems and dementia in people with MCI have produced conflicting evidence, either providing no evidence of an association (Mecca et al. 2018; Gallagher et al. 2022; Zawar et al. 2023) or an inverse association between sleep problems and dementia (Ramakers et al. 2010; Carnicelli et al. 2019). These studies in adults over the age of 50 years, who have been referred to memory clinics, were either conducted in small cohorts of people with MCI, *n* = 263 (Ramakers et al. 2010); or persons with amnestic MCI, *n* = 19 (Carnicelli et al. 2019); or specific cohorts to investigate development of AD and that are nonrepresentative for the population (Mecca et al. 2018; Gallagher et al. 2022; Zawar et al. 2023). To our knowledge, there are no published studies investigating the association between sleep duration and dementia in people with MCI.

To further investigate the role of sleep disturbances as a risk factor for disease progression to dementia in patients with MCI, we analyzed data of a population of older adults with MCI, collected as part of the English Longitudinal Study of Ageing (ELSA). We assessed whether self‐reported short (< 6 h) or long (≥ 9 h) sleep duration per night as well as poor sleep quality in older adults with MCI is associated with a higher incidence of all‐cause dementia.

## 2 Methods

### 2.1 Study Design and Setting

For this secondary data analysis, we used data from the ELSA. ELSA was set up in 2002–2003 (Wave 1) and is an ongoing panel study collected every 2 years, which is representative of persons ≥ 50 years in individual households living in England. Information on this core sample include socioeconomic status, physical and mental health, and social participation  (Steptoe et al. 2013). To maintain representative, the sample has been refreshed several times. Cross‐sectional weights were derived to adjust for non‐responding (Banks et al. 2020). Data on core members, as well as partners and other household members, were collected in face‐to‐face interviews, questionnaires, and physical examinations on a subsample at intervals of 4 years (2004–2005, 2008–2009, 2012–2013, and 2016–2017). Proxy interviews with family members were performed for individuals who had cognitive impairment and/or dementia. All participants provided informed consent, and ethical approval for each wave had been given by the National Research Ethics Committee. This secondary data analysis was approved by the Research Ethics Committee of the London School of Hygiene and Tropical Medicine (LSHTM Ethics Reference: 28555). We used anonymized interview data from Wave 4 (2008/2009) to Wave 9 (2018/2019), provided by the UK Data Service.

### 2.2 Participants

The study population was drawn from Waves 4, 6, and 8, which were the only waves to assess sleep. We included participants ≥ 50 years with MCI. To define MCI, we used a four‐way assessment, in accordance with recognized diagnostic guidelines (Winblad et al. 2004; Albert et al. 2011), based on recommendations in prior studies (Vancampfort et al. 2017; Feter et al. 2021) on the ELSA cohort. After having excluded participants with dementia and < 50 years, MCI was defined if all the following items applied: (a) Absence of self‐reported dementia or AD. (b) Subjective decline in cognition, defined by subjects rating their memory as fair or poor. (c) Preserved ability to perform daily activities. Subjects were asked if they had any difficulties in (c1) dressing and (c2) eating. Preserved functional abilities were defined if subjects stated to have no problems. (d) Objectively reduced cognitive function. This was defined as failure in at least one of three neuropsychological assessments during the main interview, which address various domains and have shown good sensitivity and specificity for detection of MCI. Assessments included orientation in time (O'Keeffe et al. 2011), episodic memory tested by a 10‐word‐recall test (Shankle et al. 2005), and verbal fluency tested by the 1‐min animal test (Radanovic et al. 2007). In the assessment of orientation, deficiency was defined by not being able to state the right month and/or right year. In the 10‐word‐recall test, the interviewer read out 10 words and the participants were asked to repeat as many words as possible immediately and about 5 min later, we calculated a score as the total number of words recalled. In the 1‐min animal test, participants were asked to name as many different animals as possible within 1 min. Both the total score of the word‐recall‐test and the number of animals were standardized into an age‐adjusted *z*‐score based on mean and standard deviation. We defined not passing as scoring < 1.0 standard deviations below the age‐adjusted *z*‐score. In Wave 6, participants were neither asked about subjective decline in cognition nor assessed for verbal fluency, these criteria were not considered when identifying MCI at Wave 6.

### 2.3 Variables

The exposure of interest was self‐reported poor overall sleep quality and self‐reported sleep duration. Both exposures were assessed in Waves 4, 6, and 8 within the main interview. We defined the first wave at which a subject's MCI status was assessed as baseline. We measured exposure status at baseline and assessed self‐reported overall sleep quality by a question adapted from the Jenkins Sleep Scale (Jenkins et al. 1988): “During the last month, how would you rate your sleep quality overall?”. We dichotomized the variable and defined poor sleep quality as rated “Fairly bad” or “Very bad.” We used a binarized score based on one single variable to maximize statistical power. Self‐reported average sleep duration per night during the preceding month was measured by an open‐ended question. We categorized sleep duration as short duration (< 6 h), normal duration (6–< 9 h), and long duration (≥ 9 h) as recommended elsewhere (Bloomberg et al. 2023). Participants with missing data for sleep assessment were excluded.

The outcome was incidence of all‐cause dementia, assessed at Waves 5–9, in the main interview. Participants were asked whether they have been diagnosed with dementia or AD since the last interview and on what date. If reported, these participants were considered as having a new diagnosis of dementia. If subjects were unable to take part in the interview, a proxy interview was performed with a relative instead. Here, dementia was assessed by the 16‐item Informant Questionnaire on Cognitive Decline in the Elderly (IQCODE) (Jorm 1994). This questionnaire asks about the subject's ability to perform different activities of daily living during the preceding 2 years and each of the 16 questions scores between 1 (*Much improved*) and 5 (*Much worse*). New dementia diagnosis was defined if participants reached ≥ 3.5 on a combined average score (Khondoker et al. 2017; Quinn et al. 2021). If a new diagnosis of dementia was self‐reported, we defined the date of diagnosis the first day of the month when dementia was diagnosed for the first time. If subjects did not remember the date as well as for the assessment by IQCODE, the date of diagnosis was defined the midpoint between the current and preceding interview.

We included several covariates that have been shown to be associated with development of dementia in MCI (Campbell et al. 2013; Cooper et al. 2015; Li et al. 2016; Baird et al. 2021) and assessed these in the interview at baseline: age (50–59, 60–69, 70–79, and ≥ 80 years), sex (male/female), ethnicity (White/non‐White), marital status (never married/married or in legal partnership/separated or divorced/widowed), education (higher education/school education/no formal qualification), smoking status (smoker/non‐ or ex‐smoker), alcohol consumption (≥ 5 days a week/less), BMI (< 18.5/18.5–< 25/≥ 25), and self‐reported physical activity (inactive/mild/moderate/vigorous). Dichotomous covariates were self‐reported history of arterial hypertension, diabetes, cardiovascular disease (CVD), loneliness, and depression. CVD was positive for any symptom among angina, heart attack, congestive heart failure, diabetes, heart murmur, abnormal heart rhythm, stroke, hypertension, and high cholesterol. Depression was measured based on the eight‐item version of the Center for Epidemiologic Studies Depression Scale (CES‐D) (Briggs et al. 2018). Subjects indicated whether they had eight specified depressive symptoms leading to a score between 0 and 8, higher scores indicating more symptoms. We defined depression as scoring ≥ 3 (Lee et al. 2021). For assessment of loneliness, we used the three‐item University of California, Los Angeles (UCLA) loneliness scale (Hughes et al. 2004): the questions “How often respondent feels lack companionship,” “How often respondent feels left out,” and “How often respondent feels isolated” are answered on a scale between 1 (*Hardly ever or never*) and 3 (*Often*). We summed the score and defined loneliness as scoring more than the median, an approach used in prior research (Rafnsson et al. 2020; Hackett et al. 2023).

### 2.4 Statistical Methods

Descriptive characteristics are presented in absolute and relative frequencies and median with interquartile range for continuous data. We calculated crude incidence rates (IRs) of dementia per 1000 person‐years (py) and IRs stratified by exposure. Rate ratios and 95% confidence intervals (CIs) of dementia incidence were calculated, comparing poor versus good sleep quality, short versus normal sleep duration, and long versus normal sleep duration. All covariates were tested for effect modification using Mantel–Haenszel methods. We also tested any association between missing versus non‐missing data in each covariate against exposure and outcome using a chi‐squared test.

We used Cox proportional hazards models based on complete cases to estimate effects of exposure on the outcome. Proportional hazards were confirmed by tests on the basis of Schoenfeld residuals. We decided a priori to include all covariates with < 10% missing observations in the primary analysis. Age was assumed a major confounder on dementia and we based the analysis on age as the primary time scale. Subjects entered observation at the age of the baseline interview and left observation at the age of dementia diagnosis, the last completed interview or at Wave 9. Subjects were excluded from the analysis if they did not complete any interviews after baseline. We fitted four models to the data: Model 1 examined the association between overall sleep quality and dementia, and Model 2 analyzed this association adjusted for covariates. Model 3 investigated the association between sleep duration and dementia, and Model 4 investigated this association adjusted for covariates. Analyses were weighted using the cross‐sectional weights provided with the ELSA data to adjust for nonresponse bias (Steptoe et al. 2013; Banks et al. 2020). We examined effect modification between exposure and sex by stratifying the primary model by sex and tested for interaction by a Wald test, as the use of weights violates the assumptions of the likelihood ratio test. We assessed interaction by age graphically using Nelson–Aalen plots. For all statistical tests we used a two‐tailed alpha level of 0.025. All statistical analyses were performed with Stata (StataCorp LLC, 2020, Stata Statistical Software: Release 16.1, College Station, TX).

### 2.5 Sensitivity Analysis

In a sensitivity analysis we fitted models to the data with all covariates included and then repeated the analysis in this reduced sample including only the exposure variable. We ran a further analysis with observation time, split in 1‐year intervals, as a confounder to exclude an independent association of the outcome with time since study entry. Taking altered criteria to define MCI into account, we also ran the primary analysis without subjects recruited at Wave 6.

Data in the ELSA interviews include three more questions referring to sleep and some researchers have used a combined score on sleep before (Song et al. 2021). Thus, we further investigated dementia incidence by a Cox proportional hazards model using a summary score of the four original qualitative questions related to sleep. Apart from the question about overall sleep quality, further questions were “How often respondent has difficulty falling asleep,” “Frequency wake up several times at night,” and “Frequency wake up feeling tired & worn out.” These questions were answered on a scale between 1 (*Not during the last month*) and 4 (*≥ 3 times a week*). We summed up the scores and divided the summary score in three categories (good quality ≥ 4–< 8, intermediate quality 8–< 12, and poor quality 12–16), with good quality being the reference category (Song et al. 2021). The Cronbach's alpha at baseline, as a measure of the internal validity, was 0.73.

## 3 Results

### 3.1 Participants

**FIGURE 1:**
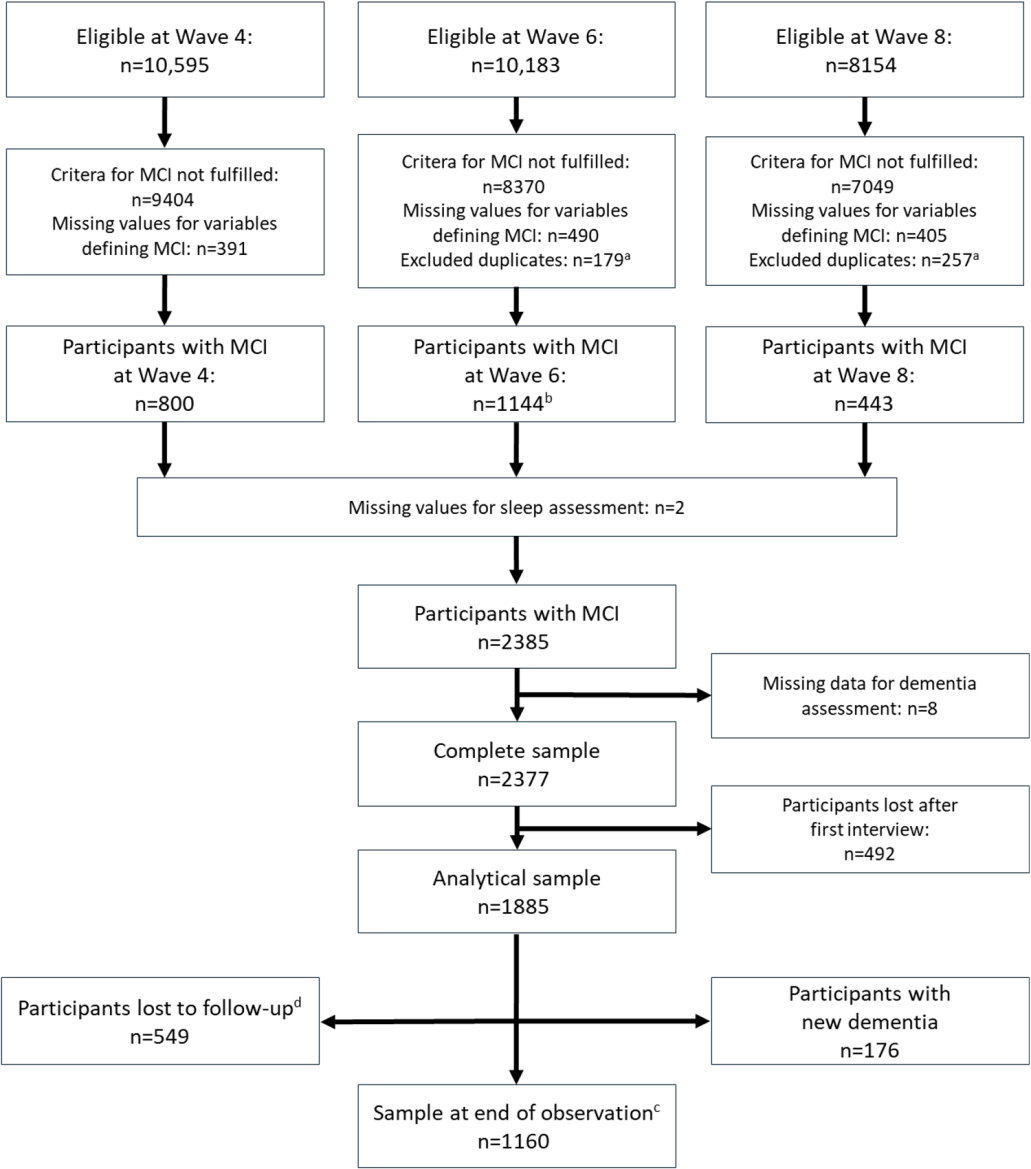
Flow diagram showing the composition of analytical sample and follow‐up. ^a^These duplicates were identified as having MCI in more than one wave. They were followed up from the first wave in which MCI was identified and not double counted. ^b^Participants recruited at Wave 6 were affected by adapted MCI diagnosis based on available information. ^c^Participants who were followed up until Wave 9. ^d^Participants who completed at least one additional interview after baseline but left observation prior to Wave 9.

A total of 2385 subjects fulfilled the inclusion criteria. After having excluded observations with missing data for dementia assessment and subjects who were lost to follow‐up immediately after the baseline interview, our analytical sample included 1885 subjects (Figure 1) with a median observation time of 5.8 years (interquartile range 2.1–6.3). Median age at baseline was 66 years (interquartile range 59–75), and 52.8% of subjects were female (*n* = 995). Among the analytical sample, 549 (29%) subjects were lost to follow‐up before Wave 9 (Figure 1).

### 3.2 Descriptive Data

One quarter of participants, 24.7% (466/1885), reported poor overall sleep quality. People with poor versus good sleep quality were more likely to be older; female; White; nonsmoker; and more frequently reported to suffer from any CVD, depression, and loneliness. They were mostly in the age group 60–69 years, married, without formal education, and reported moderate physical activity (Table 1). Regarding self‐reported average sleep duration per night, 17.6% (331/1879) mentioned short sleep duration and 7.5% (141/1879) mentioned long duration. Among participants with short sleep duration, 63.4% (210/331) reported poor overall sleep quality, whereas only 5.7% (8/141) among those sleeping ≥ 9 h per night stated poor sleep quality. Amid individuals with normal sleep duration, 17.4% (245/1407) announced overall poor sleep quality.

**TABLE 1:** Total and relative frequencies for the following variables: (1) overall sleep quality and (2) average sleep duration per night, by dementia outcome and each covariate. Missing observations are excluded.

		Overall sleep quality (n = 1885)					Average sleep duration per night (n = 1,879)				
Variable	Characteristics	Total *N*; %^a^	Good Total *N*; %^a^	Poor Total *N*; %^a^	*p*‐value^b^	*N* (per variable)	Total *N*; %^a^	< 6 h Total *N*; %^a^	6–9 h Total *N*; %^a^	≥ 9 h Total *N*; %^a^	*p*‐value^b^
All‐cause dementia reported		176 (100)	146 (83.0)	30 (17.1)	0.013		175 (100)	20 (11.4)	118 (67.4)	37 (21.1)	< 0.01
Age group (*n* = 1885)	50–59	472 (25.0)	322 (22.7)	150 (32.2)	< 0.01	*n* = 1879	470 (25.0)	85 (25.7)	367 (26.1)	18 (12.8)	< 0.01
	60–69	702 (37.2)	531 (37.4)	171 (36.7)			702 (37.4)	122 (36.9)	545 (38.7)	35 (24.8)	
	70–79	494 (26.2)	393 (27.7)	101 (21.7)			490 (26.1)	89 (26.9)	350 (24.9)	51 (36.2)	
	≥ 80	217 (11.5)	173 (12.2)	44 (9.4)			217 (11.6)	35 (10.6)	145 (10.3)	37 (26.2)	
Sex (*n* = 1885)	Male	890 (47.2)	718 (50.6)	172 (36.9)	< 0.01	*n* = 1879	887 (47.2)	127 (38.4)	697 (49.5)	63 (44.7)	< 0.01
	Female	995 (52.8)	701 (49.4)	294 (63.1)			992 (52.8)	204 (61.3)	710 (50.5)	78 (55.3)	
Ethnicity (*n* = 1884)	White	1750 (92.9)	1322 (93.2)	428 (91.6)	0.313	*n* = 1878	1744 (92.9)	294 (88.8)	1315 (93.5)	135 (95.7)	0.004
	Non‐White	134 (7.1)	96 (6.8)	38 (8.2)			134 (7.1)	37 (11.2)	91 (6.5)	6 (4.3)	
Marital status (*n* = 1885)	Never	143 (7.6)	96 (6.8)	47 (10.1)	0.004	*n* = 1879	143 (7.6)	30 (9.1)	108 (7.7)	5 (3.6)	< 0.01
	Married/legal partner	1169 (62.0)	908 (64.0)	261 (56.0)			1167 (62.1)	185 (55.9)	906 (64.4)	76 (53.9)	
	Separated/divorced	241 (12.8)	167 (11.8)	74 (15.9)			239 (12.7)	49 (14.8)	181 (12.9)	9 (6.4)	
	Widowed	332 (17.6)	248 (17.5)	84 (18.0)			330 (17.6)	67 (20.2)	212 (15.1)	51 (36.2)	
Education (*n* = 1845)	Higher education	409 (22.2)	336 (24.2)	73 (16.1)	0.001	*n* = 1839	409 (22.2)	58 (17.8)	329 (23.9)	22 (16.1)	0.001
	School education	703 (38.1)	526 (37.8)	177 (39.0)			700 (38.1)	122 (37.4)	534 (38.8)	44 (32.1)	
	No qualification	733 (39.7)	529 (38.0)	204 (44.9)			730 (39.7)	146 (44.8)	513 (37.3)	71 (51.8)	
Smoking (*n* = 1212)	Ex/never	913 (75.3)	695 (77.4)	218 (69.4)	0.005	*n* = 1208	911 (75.4)	164 (72.9)	684 (76.1)	63 (75.0)	0.607
	Current	299 (24.7)	203 (22.6)	96 (30.6)			297 (24.6)	61 (27.1)	215 (23.9)	21 (25.0)	
Alcohol (*n* = 1289)	≥ 5 days a week	283 (22.0)	223 (22.6)	60 (19.9)	0.317	*n* = 1287	282 (21.9)	41 (19.2)	221 (22.2)	20 (25.6)	0.442
	Less	1006 (78.0)	764 (77.4)	242 (80.1)			1005 (78.1)	173 (80.8)	774 (77.8)	58 (74.4)	
Hypertension (*n* = 1815)	No	1093 (60.2)	839 (61.4)	254 (56.7)	0.079	*n* = 1809	1098 (60.2)	194 (59.9)	817 (60.4)	78 (59.1)	0.951
	Yes	722 (39.8)	528 (38.6)	194 (43.3)			720 (39.8)	130 (40.1)	536 (39.6)	54 (40.9)	
											
Diabetes (*n* = 1879)	No	1650 (87.8)	1254 (88.7)	396 (85.2)	0.044	*n* = 1873	1645 (87.8)	291 (88.2)	1237 (88.2)	117 (83.0)	0.187
	Yes	229 (12.2)	160 (11.3)	69 (14.8)			228 (12.2)	39 (11.8)	165 (11.8)	24 (17.0)	
CVD (*n* = 1523)	No	362 (23.8)	265 (23.5)	97 (24.6)	0.669	*n* = 1518	359 (23.7)	72 (26.0)	270 (24.0)	17 (14.9)	0.057
	Yes	1,161 (76.2)	863 (76.5)	298 (75.4)			1159 (76.4)	205 (74.0)	857 (76.0)	97 (85.1)	
BMI^c^ (*n* = 1249)	< 18.8	10 (0.8)	7 (0.7)	3 (1.0)	0.607	*n* = 1246	10 (0.8)	2 (0.1)	6 (0.6)	2 (2.1)	0.597
	18.5–< 25	307 (24.6)	239 (25.2)	68 (22.6)			306 (24.6)	48 (24.1)	237 (24.9)	21 (22.3)	
	≥ 25	932 (76.6)	702 (74.1)	230 (76.4)			930 (74.6)	149 (74.9)	710 (74.5)	71 (75.5)	
Depression (*n* = 1870)	No	1363 (72.9)	1159 (82.1)	204 (44.5)	< 0.01	*n* = 1864	1360 (73.0)	182 (55.3)	1069 (76.7)	109 (77.3)	< 0.01
	Yes	507 (27.1)	253 (17.9)	254 (55.5)			504 (27.0)	147 (44.7)	325 (23.3)	32 (22.7)	
Loneliness (*n* = 1629)	No	19 (1.2)	14 (1.1)	5 (1.3)	0.817	*n* = 1626	19 (1.2)	4 (1.4)	14 (1.1)	1 (0.9)	0.878
	Yes	1610 (98.8)	1223 (98.9)	387 (98.7)			1607 (98.8)	275 (98.6)	1220 (98.9)	112 (99.1)	
Physical activity (*n* = 1884)	Inactive	179 (9.5)	116 (8.2)	63 (13.6)	< 0.01	*n* = 1878	177 (9.4)	38 (11.5)	124 (8.8)	15 (10.6)	0.007
	Mild	395 (21.0)	280 (19.7)	115 (24.7)			393 (20.9)	72 (21.7)	283 (20.1)	38 (27.0)	
	Moderate	856 (45.4)	662 (46.7)	194 (41.7)			854 (45.5)	153 (46.2)	631 (44.9)	70 (49.7)	
	Vigorous	454 (24.1)	361 (25.4)	93 (20.0)			454 (24.2)	68 (20.5)	368 (26.2)	18 (12.8)	

^a^ Column percentage.

^b^ *p*‐value for chi‐squared test.

^c^ BMI is only available for subjects included at Waves 4 and 6.

Covariates with > 10% missing observations were smoking (673, 36%), alcohol consumption (596, 32%), CVD (362, 19%), BMI (636, 34%), and loneliness (256, 14%). Data on BMI only were available from Waves 4 and 6. Other covariates with missing values were hypertension (70, 3.7%), depression (15, 0.8%), and diabetes (6, 0.3%). For all covariates missing and non‐missing observations were evenly distributed between good and poor sleep quality as well as short, normal, and long sleep duration (Table 3). Participants who lost to follow‐up after the baseline assessment tended to be older, widowed, less educated, more likely to be underweight and physically inactive, and have longer sleep duration (Table 7).

**TABLE 2:** Effect of overall sleep quality and average sleep duration per night on dementia incidence using Cox proportional hazards models.

Variable	Characteristics	Model 1^a^ HR (95% CI) *n* = 1885	Model 2^b^ HR (95% CI) n = 1793	Model 3^c^ HR (95% CI) n = 1879	Model 4^d^ HR (95% CI) n = 1787
Overall sleep quality	Good	1.00	1.00	—	—
	Poor	0.73 (0.47–1.13) *p* = 0.162	0.68 (0.41–1.12) *p* = 0.133	—	—
Average sleep duration per night	6–9 h	—	—	1.00	1.00
	< 6 h	—	—	0.89 (0.53–1.49) *p* = 0.658	0.94 (0.55–1.61) *p* = 0.824
	≥ 9 h	—	—	2.46 (1.65–3.65) *p* < 0.01	2.57 (1.62–4.02) *p* < 0.01

^a^ Included variables: age‐adjusted overall sleep quality.

^b^ Included variables: age‐adjusted overall sleep quality, sex, ethnicity, marital status, education, hypertension, diabetes, depression, and physical activity.

^c^ Included variables: age‐adjusted average sleep duration per night.

^d^ Included variables: age‐adjusted average sleep duration per night, sex, ethnicity, marital status, education, hypertension, diabetes, depression, and physical activity.

**TABLE 3:** Association between missing and non‐missing observations within each variable with missing observations.

		Overall sleep quality (*n* = 1885)				Average sleep duration per night (*n* = 1879)				
Variable	Characteristics	Total *N*; %^a^	Good Total *N*; %^a^	Poor Total *N*; %^a^	*p*‐value^b^	Total *N*; %^a^	< 6h Total *N*; %^a^	6–9 h Total *N*; %^a^	≥ 9h Total *N*; %^a^	*p*‐value^b^
Ethnicity	non‐missing	1884 (99.6)	1418 (99.9)	466 (100)	0.566	1878 (99.9)	331 (100)	1406 (99.9)	141 (100)	0.846
	missing	1 (0.1)	1 (0.1)	0		1 (0.1)	0	1 (0.1)	0	
Education	non‐missing	1845 (97.9)	1391 (98.0)	454 (97.4)	0.434	1839 (97.9)	326 (98.5)	1376 (97.8)	137 (97.2)	0.611
	missing	40 (2.1)	28 (2.0)	12 (2.6)		40 (2.1)	5 (1.5)	31 (2.2)	4 (2.8)	
Smoking	non‐missing	1212 (64.3)	989 (63.3)	314 (67.4)	0.109	1208 (64.3)	225 (68.0)	899 (63.9)	84 (59.6)	0.181
	missing	673 (35.7)	521 (36.7)	152 (32.6)		671 (35.7)	106 (32.0)	508 (36.1)	57 (40.4)	
Alcohol	non‐missing	1289 (68.4)	987 (69.6)	302 (64.8)	0.056	1287 (68.5)	214 (64.7)	995 (70.7)	78 (55.3)	< 0.01
	missing	596 (31.6)	432 (30.4)	164 (35.2)		592 (31.5)	117 (35.4)	412 (29.9)	63 (44.7)	
Hypertension	non‐missing	1815 (96.3)	1367 (96.3)	448 (96.1)	0.844	1809 (96.3)	324 (97.9)	1353 (96.2)	132 (93.6)	0.074
	missing	70 (3.7)	52 (3.7)	18 (3.9)		70 (3.7)	7 (2.1)	54 (3.8)	9 (6.4)	
Diabetes	non‐missing	1879 (99.7)	1414 (99.7)	465 (99.8)	0.647	1873 (99.7)	330 (99.7)	1402 (99.6)	141 (100)	0.774
	missing	6 (0.3)	5 (0.4)	1 (0.2)		6 (0.3)	1 (0.3)	5 (0.4)	0	
CVD	non‐missing	1523 (80.8)	1128 (79.5)	395 (84.8)	0.012	1518 (80.8)	227 (83.7)	1127 (80.1)	114 (80.9)	0.329
	missing	362 (19.2)	291 (20.5)	71 (15.2)		361 (19.2)	54 (16.3)	280 (19.9)	27 (19.2)	
BMI^c^	non‐missing	1249 (66.3)	948 (66.8)	301 (64.6)	0.380	1246 (66.3)	199 (60.1)	953 (67.7)	94 (66.7)	0.031
	missing	636 (33.7)	471 (33.2)	165 (33.4)		633 (33.7)	132 (39.9)	454 (32.3)	47 (33.3)	
Depression	non‐missing	1870 (99.2)	1412 (99.5)	458 (98.3)	0.010	1864 (99.2)	329 (99.4)	1394 (99.1)	141 (100)	0.456
	missing	15 (0.8)	7 (0.5)	8 (1.7)		15 (0.8)	2 (0.6)	13 (0.9)	0	
Loneliness	non‐missing	1629 (86.4)	1237 (87.2)	392 (84.1)	0.095	1626 (86.5)	279 (84.3)	1234 (87.7)	113 (80.1)	0.018
	missing	256 (13.6)	182 (12.8)	74 (15.9)		253 (13.5)	52 (15.7)	173 (12.3)	28 (19.9)	
Physical activity	non‐missing	1884 (99.9)	1419 (100)	465 (99.8)	0.081	1878 (99.9)	331 (100)	1406 (99.9)	141 (100)	0.846
	missing	1 (0.1)	0	1 (0.2)		1 (0.1)	0	1 (0.1)	0	

^a^ Column percentage.

^b^ *p*‐value for chi‐squared test.

^c^ BMI is only available for subjects included at Waves 4 and 6.

### 3.3 Outcome Data

Among the analytical sample, 176 subjects had developed self‐reported all‐cause dementia. The diagnosis was based on a proxy interview in 84 cases (48%). Dementia had a cumulative incidence of 9.3% and an IR of 17.9 per 1000 py (95% CI 15.4–20.7).

### 3.4 Main Results

**FIGURE 2:**
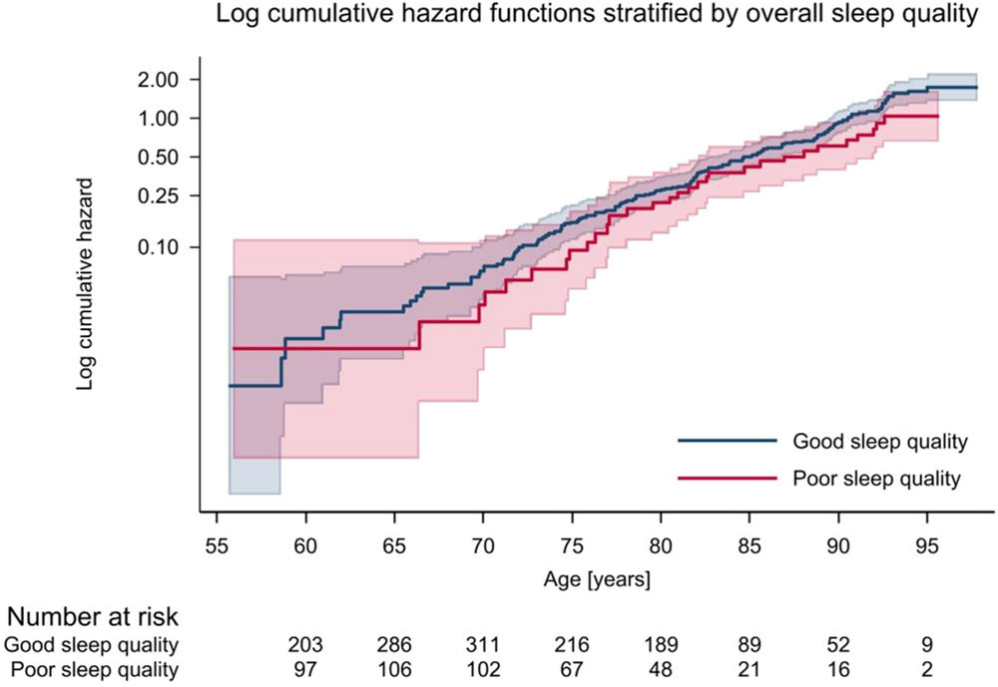
Log cumulative hazard functions with accompanying 95% confidence intervals, stratified by overall sleep quality. A test of the proportional hazards assumption based on Schoenfeld residuals indicated no violation for exposure variable (global *p* = 0.975, estat phtest in Stata).

**FIGURE 3:**
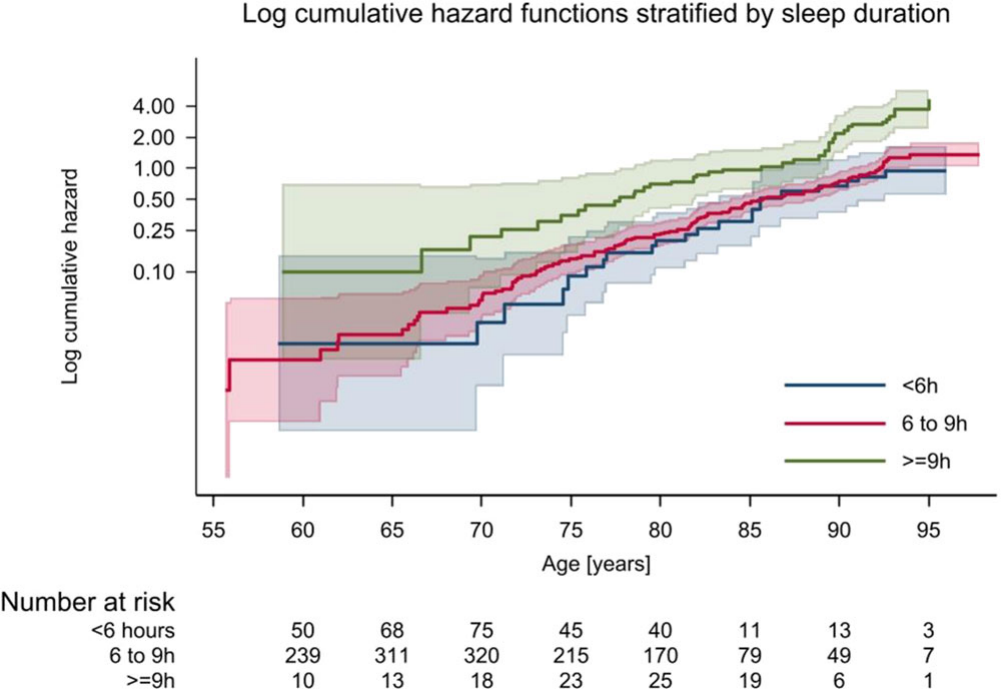
Log cumulative hazard functions with accompanying 95% confidence intervals, stratified by average sleep duration per night. A test of the proportional hazards assumption based on Schoenfeld residuals indicated no violation for exposure variable (global *p *= 0.988, estat phtest in Stata).

We fitted four Cox proportional hazards models to the data (Table 2). There was weak evidence that self‐reported poor overall sleep quality at baseline is associated with reduced dementia incidence (age‐adjusted hazard ratio [HR] 0.73, 95% CI 0.47–1.13). Controlling for sex, ethnicity, marital status, education, hypertension, diabetes, depression, and physical activity did not reveal any statistically significant association (HR 0.68, 95% CI 0.41–1.12). There was little evidence that baseline self‐reported short sleep duration, compared to normal duration, was associated with dementia incidence before or after adjustment for sex, ethnicity, marital status, education, hypertension, diabetes, depression, and physical activity (age‐adjusted HR 0.89 [95% CI 0.53–1.49], adjusted HR 0.94 [95% CI 0.55–1.61]). Self‐reported sleep duration ≥ 9 h, compared to normal sleep duration, showed an increased dementia incidence (age‐adjusted HR 2.46, 95% CI 1.65–3.65). Evidence of this association remained strong after adjustment for sex, ethnicity, marital status, education, hypertension, diabetes, depression, and physical activity (HR 2.57, 95% CI 1.62–4.02). There was no evidence of an effect modification by age (Figures 2 and 3) or sex (Table 4).

**TABLE 4:** Effect of overall sleep quality and average sleep duration per night on dementia, stratified by sex and using Cox proportional hazards model including an interaction factor between exposure and sex.

HR for exposure, stratified by sex		HR for males (95% CI)	HR for females (95% CI)	*p*‐value^c^
Overall sleep quality^a^	Good	1.00 (reference)		0.319
	Poor	0.68 (0.41–1.53)	0.69 (0.39–1.24)	
Average sleep duration per night^b^	6–9 h	1.00 (reference)		0.536
	< 6 h	1.19 (0.55–2.57)	0.78 (0.38–1.59)	
	≥ 9 h	3.47 (1.92–6.26)	2.08 (1.14–3.81)	

^a^ Included variables: age‐adjusted overall sleep quality, sex, ethnicity, marital status, education, hypertension, diabetes, depression, and physical activity.

^b^ Included variables: age‐adjusted overall sleep quality, sex, ethnicity, marital status, education, hypertension, diabetes, depression, and physical activity.

^c^ *p*‐value of adjusted Wald test comparing model with and without interaction term.

**TABLE 5:** Sensitivity analysis with effect of overall sleep quality and average sleep duration per night on dementia incidence using Cox proportional hazards models (all covariates included).

Variable	Characteristic	Model 5^a^ HR (95% CI) *n* = 607	Model 5b^b^ HR (95% CI) *n* = 607	Model 6^c^ HR (95% CI) *n* = 606	Model 6b^d^ HR (95% CI) *n* = 606	Model 7^e^ HR (95% CI) *n* = 1787	Model 8^f^ HR (95% CI)‍ *n* = 934
Overall sleep quality	Good	1.00	1.00	—	—	—	1.00
	Poor	0.57 (0.22–1.44) *p* = 0.235	0.90 (0.38–1.99) *p* = 0.740	—	—	—	0.43 (0.22–0.87) *p* = 0.018
Average sleep duration per night	6–9 h	—	—	1.00	1.00	—	1.00^g^
	< 6 h	—	—	1.03 (0.43–2.44) *p* = 0.953	1.04 (0.39–2.73) *p* = 0.942	—	0.89 (0.44–1.79) *p* = 0.746
	≥ 9 h	—	—	1.22 (0.29–5.07) *p* = 0.787	1.60 (0.39–2.73) *p* = 0.383	—	3.07 (1.57–5.99) *p* < 0.01
Sleep summary score	Good sleep quality	—	—	—	—	1.00	1.00^h^
	Intermediate sleep quality	—	—	—	—	0.58 (0.40–0.85) *p* < 0.01	0.50 (0.30–0.83) *p* < 0.01
	Poor sleep quality	—	—	—	—	0.71 (0.42–1.18) *p* = 0.187	0.45 (0.21–0.93) *p* = 0.031

^a^ Included variables: age‐adjusted overall sleep quality, sex, ethnicity, marital status, education, hypertension, diabetes, depression, physical activity, loneliness, BMI, alcohol consumption, smoking, and CVD.

^b^ Age‐adjusted overall sleep quality only included (no other covariates), but model run in reduced sample of Model 5 (*n* = 607).

^c^ Included variables: age‐adjusted average sleep duration per night, sex, ethnicity, marital status, education, hypertension, diabetes, depression, physical activity, loneliness, BMI, alcohol consumption, smoking, and CVD.

^d^ Age‐adjusted average sleep duration per night only included (no other covariates), but model run in reduced sample of Model 6 (*n* = 606).

^e^ Included variables: sleep summary score, sex, ethnicity, marital status, education, hypertension, diabetes, depression, and physical activity.

^f^ Model including only subjects recruited at Waves 4 and 8; included variables: age‐adjusted exposure, sex, ethnicity, marital status, education, hypertension, diabetes, depression, and physical activity.

^g^ *n* = 932, due to missing values of the variable average sleep duration.

^h^ *n* = 905, due to missing values within variables assessing sleep.

### 3.5 Sensitivity Analysis

We performed a sensitivity analysis using a model for dementia incidence in poor compared to good sleep quality, having added all other covariates (Model 5 = Model 2 + smoking, alcohol, CVD, BMI, loneliness; Table 5). In this model HR only changed marginally from 0.68 to 0.57, without evidence for an effect of poor sleep quality on dementia incidence (95% CI 0.22–1.44, *n* = 607). We also tested the effect of sleep duration in a model with all covariates included (Table 5). For short sleep duration the HR increased from 0.94 to 1.03 (95% CI 0.43–2.44); for long sleep duration the HR declined from 2.57 to 1.22 (95% CI 0.29–5.07) and evidence that long sleep duration is associated with dementia in people with MCI was weakened (*p* = 0.787, *n* = 606). The effect of long sleep duration altered in this reduced complete case sample with only exposure included (HR 1.60, 95% CI 0.39–2.73; Table 5). Further, there was no evidence that time since study entry confounds the association between exposures and outcome (Table 6). Applying the summary sleep quality score, 36% (673/1885) of subjects reported good quality, 41.5% (783/1885) intermediate quality, and 23% (429/1885) poor quality sleep. In a model controlled for sex, ethnicity, marital status, education, hypertension, diabetes, depression, and physical activity (*n* = 1787; Table 5), there was reasonable evidence that subjects with intermediate sleep quality, compared to subjects with good quality, had a decreased hazard for dementia (HR 0.58, 95% CI 0.40–0.85). Participants with poor quality, compared to good quality, showed a similar result, however, with weakened evidence for a difference (HR 0.71, 95% CI 0.42–1.18). A model without subjects recruited from Wave 6 (Model 8) showed more extreme HRs for poor sleep quality (HR 0.43, 95% CI 0.22–0.87) and long sleep duration (HR 3.07, 95% CI 1.57–5.99) (Table 5). Point estimates were within the 95% CIs of the primary model.

**TABLE 6:** Sensitivity analysis with effect of overall sleep quality and average sleep duration per night on dementia incidence, controlled for time since study entry.

Variable	Characteristic	Model 1^a^ HR (95% CI)	Model 3^b^ HR (95% CI)	Model 9^c^ HR (95% CI)	Model 10^d^ HR (95% CI)
Overall sleep quality	Good	1.00	—	1.00	—
	Poor	0.73 (0.47–1.13)	—	0.74 (0.48–1.14)	—
Average sleep duration per night	6–9 h	—	1.00	—	1.00
	< 6 h	—	0.89 (0.53–1.49)	—	0.87 (0.52–1.46)
	≥ 9 h	—	2.46 (1.65–3.65)	—	2.39 (1.62–5.54)

^a^ Cox proportional hazards model including age‐adjusted overall sleep quality.

^b^ Cox proportional hazards model including age‐adjusted average sleep duration per night.

^c^ Cox proportional hazards model including overall sleep quality with age as primary time scale and time since study entry, split in intervals of 1 year, added to model as covariate.

^d^ Cox proportional hazards model including average sleep duration per night with age as primary time scale and time since study entry, split in intervals of 1 year, added to model as covariate.

**TABLE 7:** Totals and relative frequencies of characteristics of subjects included in the study, compared to subjects who were lost to follow‐up after the baseline interview, and excluded from the survival analysis.

Variable	Characteristic	Total *N*; %^a^	Included subjects Total *N*; %^a^	Loss to FU after baseline interview Total *N*; %^a^	*p*‐value^b^
Total of subjects being eligible		2379 (100)	1885 (79.2)	494 (20.8)	
Overall sleep quality (*n* = 2,378)	Good	1800 (75.7)	1419 (75.3)	381 (77.3)	0.356
	Poor	578 (24.3)	466 (24.7)	112 (22.7)	
Sleep duration (*n* = 2,370)	< 6h	418 (17.6)	331 (17.6)	87 (17.7)	0.003
	6–9h	1751 (73.9)	1407 (74.9)	344 (70.1)	
	≥ 9h	201 (8.5)	141 (7.5)	60 (12.2)	
Age group (*n* = 2379)	50–59	576 (24.1)	482 (25.0)	104 (21.1)	0.000
	60–69	834 (35.1)	702 (37.2)	132 (26.7)	
	70–79	626 (26.3)	494 (26.2)	132 (26.7)	
	80–89	294 (12.4)	197 (10.5)	97 (19.6)	
	≥ 90	49 (2.1)	20 (1.1)	29 (5.9)	
Sex (*n* = 2379)	Male	1119 (47.0)	890 (47.2)	229 (46.4)	0.734
	Female	1260 (53.0)	995 (52.8)	265 (53.6)	
Ethnicity (*n* = 2377)	White	2209 (92.9)	1750 (92.9)	459 (93.1)	0.868
	Non‐White	168 (7.1)	134 (7.1)	34 (6.9)	
Marital status (*n* = 2379)	Never	193 (8.1)	143 (7.6)	50 (10.1)	0.000
	Married/legal partner	1430 (60.1)	1169 (62.0)	261 (52.8)	
	Separated/divorced	294 (12.4)	241 (12.8)	53 (10.7)	
	Widowed	462 (19.4)	332 (17.6)	130 (26.3)	
Education (*n* = 2320)	Higher education	486 (21.0)	409 (22.1)	77 (16.2)	0.000
	School education	859 (37.0)	703 (38.1)	156 (32.8)	
	No qualification	975 (42.0)	733 (39.7)	242 (51.0)	
Smoking (*n* = 1515)	Ex/Never	1125 (74.3)	913 (75.3)	212 (70.0)	0.056
	Current	390 (25.7)	299 (24.7)	91 (30.0)	
Alcohol consumption (*n* = 1539)	≥ 5 days a week	335 (21.8)	283 (22.0)	52 (20.8)	0.685
	Less	1204 (78.2)	1006 (78.0)	198 (79.2)	
BMI (*n* = 1445)	< 18.8	16 (1.1)	10 (0.8)	6 (3.1)	0.001
	18.5–< 25	369 (25.5)	307 (24.6)	62 (31.6)	
	≥ 25	1060 (73.4)	932 (74.6)	128 (65.3)	
Hypertension (*n* = 2279)	No	1362 (59.8)	1093 (60.2)	269 (58.0)	0.379
	Yes	917 (40.2)	722 (39.8)	195 (42.0)	
Diabetes (*n* = 2369)	No	2070 (87.4)	1650 (87.8)	420 (85.7)	0.213
	Yes	299 (12.6)	229 (12.2)	70 (14.3)	
					
CVD (*n* = 1926)	No	442 (23.0)	362 (23.8)	80 (19.9)	0.096
	Yes	1484 (77.1)	1161 (76.2)	323 (80.2)	
Depression (*n* = 2361)	No	1702 (72.1)	1363 (72.9)	339 (69.0)	0.091
	Yes	659 (27.9)	507 (27.1)	152 (31.0)	
Loneliness (*n* = 1969)	No	26 (1.3)	19 (1.2)	7 (2.1)	0.190
	Yes	1943 (98.7)	1610 (98.8)	333 (97.9)	
Physical activity (*n* = 2378)	Inactive	271 (11.4)	179 (9.5)	92 (18.6)	0.000
	Mild	524 (22.0)	395 (21.0)	129 (26.1)	
	Moderate	1061 (44.6)	856 (45.4)	205 (41.5)	
	Vigorous	522 (22.0)	454 (24.1)	68 (13.8)	

^a^ Column percentages.

^b^ *p*‐value for chi‐squared test.

## 4 Discussion

### 4.1 Summary of Findings

We investigated the relationship between baseline self‐reported overall sleep quality, self‐reported sleep duration, and hazard of all‐cause dementia in a cohort of participants with investigator‐defined MCI. Age‐adjusted Cox‐regression models, controlled for multiple confounders, revealed weak evidence for poor sleep quality (HR 0.68, 95% CI 0.41–1.12, *p* = 0.133) and little evidence for short sleep duration of < 6 h per night (HR 0.94, 95% CI 0.55–1.61, *p* = 0.824) altering the hazard of all‐cause dementia. Participants with long average sleep duration (≥ 9 h per night) had an increased age‐adjusted hazard for all‐cause dementia compared to normal sleep duration (HR 2.57, 95% CI 1.62–4.02, *p* < 0.01).

### 4.2 Strengths and Weaknesses of the Study

To our best knowledge, this is the first study analyzing the effect of sleep length and quality on conversion from MCI to all‐cause dementia in the ELSA cohort. ELSA is a nation‐wide population‐based study and representative for people ≥ 50 years in England. By pooling patients with MCI included from three waves, we enhanced our sample size considerably and we incorporated important potential confounders.

The definition of the cohort, exposure, and outcome, which takes into account self‐reported information only, is an important limitation of our study. Diagnosis of MCI is complex, evolving (Petersen et al. 2018), and requires clinician input. We used a traditional definition of MCI (Winblad et al. 2004; Albert et al. 2011; Petersen et al. 2014) to ensure comparability with similar research. Misclassification of MCI through using this traditional definition and our data‐driven approach may have caused selection bias. For example, due to using incomplete criteria in Wave 6, people without MCI may have been included, diluting the association between MCI and dementia outcomes. MCI is classified in subtypes based on the affected memory domain (amnestic vs. non‐amnestic MCI) (Petersen 2004) with amnestic MCI being more likely to progress to AD (Yaffe et al. 2006). Relying on the information available from ELSA interviews, we were not able to stratify the analysis between MCI subtypes. Our findings therefore summarize the association for all MCI types and may mask differences in the direction or magnitude of the association between MCI subtypes. Exposure measurement relied on self‐reported information from interview data without using objective measures, such as polysomnography and actigraphy. This, along with a limited time frame of 1 month covered by the interview questions, may lead to unpredictable information bias. Furthermore, our primary sleep quality variable used binarized data from a single question measuring sleep quality. We tested an alternative three‐category variable based on four items from the Jenkins scale (Jenkins et al. 1988). This resulted in similar HRs to our binary sleep quality variable when both intermediate and poor sleep quality were compared to good sleep quality, supporting our findings. We based the outcome on self‐reporting of clinician‐diagnosed dementia or information from proxy interviews. This approach is susceptible to information bias, and we are aware that dementia is very probably underrepresented in ELSA (Ma et al. 2020). Assuming that the bias is the same in people with and without sleep disturbance, this information bias might reduce the magnitude of the observed association. The effect of sleep on further cognitive decline may vary between dementia subtypes and has been hypothesized especially for AD (Casagrande et al. 2022). However, the nature of the data did not allow us to differentiate between forms of dementia with distinct underlying pathologies and may mask this association.

We adjusted the model for important confounders that we could measure in ELSA. Information on further relevant confounders and mediators (objective hearing impairment, use of sleep medication, sleep apnea) was not collected within the analyzed waves. Unmeasured confounding and measurement error of confounders, resulting in residual confounding, lowered the ability to sufficiently control for confounding. In particular, the data we used do not include information on biomarkers, such as apolipoprotein E genotype (Li et al. 2016), and we were not able to stratify the hazards by biomarkers, that are known to be predictors of dementia risk.

A large proportion of people (*n* = 1286) were excluded from the cohort because they had missing observations for the variables used to define MCI and up to 5% of people with MCI were excluded from the primary analysis because of missing covariate data. This complete case methodology assumes that the independent associations we estimated were the same in the complete case cohort as the full cohort. This assumption would be incorrect if the risk of dementia, conditional on the covariates, differs in the full cohort compared to the complete case sample (White and Carlin 2010). The unadjusted model in the reduced complete case sample of the sensitivity analysis showed a smaller association between long sleep duration and dementia, compared to the primary analysis. While this difference may have arisen by chance alone, it also could reflect selection bias introduced by the complete case approach. Due to many missing values, we did not control for some confounders in the main analysis, yet we cannot exclude biased adjusted HRs if the outcome is conditional on these unobserved covariates.

Loss to follow‐up after the baseline interview may have caused attrition bias, as we therefore had to exclude a significant number of subjects considered eligible (494/2379, 20.8%). To address potential loss to follow‐up due to incident dementia, we used proxy‐based interviews if participants could no longer take part in interviews. However, information in ELSA do not cover adequate reasons for dropping out from the study and we could not fully exclude informative censoring.

Given dementia diagnoses are commonly delayed, this analysis may be subject to reverse causation. In this context, we could not differentiate whether sleep modifies the conversion from MCI to dementia or appears as an early symptom of a dementia syndrome.

### 4.3 Strengths and Weaknesses in Relation to Other Studies

To our knowledge, this is the first analysis to specifically investigate the effect of sleep duration on dementia incidence in MCI and we found evidence that long sleep duration ≥ 9 h is associated with an increased hazard of dementia. Previous observational studies revealed that the average sleep duration is longer in AD compared to MCI (Basta et al. 2019), excessive sleep is more frequent in dementia than in MCI (Gallagher et al. 2022), and an increased sleep duration over time is associated with a higher incidence risk of MCI (Wang et al. 2021). Our finding of excessive sleep duration in persons with MCI being associated with disease progression complements these prior results.

Regarding the influence of sleep quality on dementia, point estimates from previous investigations using a range of definitions of sleep quality suggest both positive and negative associations, often associated with weak statistical evidence. We found weak evidence of lower risk of conversion to dementia in people with poor sleep quality, which is supported by two studies. First, a cohort of MCI patients with sleep problems in a Dutch memory clinic showed strong statistical evidence of a lower risk for AD, compared to patients without sleep problems, after an average follow‐up time of 5.4 years (odds ratio [OR] 0.35, 95% CI 0.20–0.62) (Ramakers et al. 2010). The diagnosis of dementia was made objectively by two experts in a sample of 263 patients and adjustment for confounding included age sex and education. Second, a study retrospectively analyzed the conversion from MCI to AD in a cohort from the Alzheimer's Disease Neuroimaging Initiative (ADNI), providing weak evidence for a lower hazard of conversion to AD in MCI patients with self‐reported sleep disturbances compared to absence of sleep disturbance (HR 0.87, 95% CI 0.64–1.18) (Mecca et al. 2018). Sleep problems were measured subjectively and objectively by sleep scales and actigraphy in 308 patients observed for 16–22 months. In contrast, weak evidence was detected that a history of any sleep disorders is associated with AD incidence in 667 people with MCI (OR 1.74, 95% CI 0.94–3.23)  (Choe et al. 2022). The authors controlled for the apolipoprotein E genotype and other important confounders, but the history of sleep problems was not based on a validated scale. Another analysis from the National Alzheimer's Coordinating Center (NACC) database suggested no association between risk of AD in MCI patients with sleep disturbance (HR 1.01, 95% CI 0.92–1.10) (Zawar et al. 2023). This analysis was based on a sample of 12,281 patients, which is much higher than our sample. Presence or absence of sleep disturbance was measured by one interview question. However, only age, sex, race, and education were included as confounders and the authors decided over MCI and dementia diagnosis using a single cognitive test, the Montreal Cognitive Assessment score, probably resulting in a distinct study population.

### 4.4 Meaning of the Study

In a meta‐analysis, sleep length > 9 and ≤ 5 h per night have been related to poor cognitive outcome (Lo et al. 2016) and a cross‐sectional analysis provided some evidence that increased levels of inflammatory biomarkers in long‐sleepers induce cognitive impairment (Prather et al. 2015). Extended sleep may either reflect a physiologically prolonged need for sleep or could be in parts attributable to poor sleep quality as a compensation. Moreover, self‐reported long sleep duration may be a proxy for extended time in bed due to comorbidities. Recently, a subpopulation among long sleepers was hypothesized to significantly contribute to the U‐shaped curve: people with a physiologically higher need of sleep may restrict their sleep due to social norms and therefore suffer from chronic sleep deprivation (Balkin et al. 2024). In our study, most long sleepers reported good overall sleep quality suggesting a physiologic need for prolonged sleep. This suggests that long sleep duration might serve as an easily measurable predictor for dementia conversion in persons with MCI. Although the underlying cause is not understood well, clinicians should intend to improve sleep in persons with MCI.

Disturbed sleep is thought to modify the effects of neurotoxin aggregation in the central nervous system, leading to inflammatory processes and hence mediates cognitive decline and dementia (H. Kim et al. 2022; R. Kim et al. 2024). However, in combination with previous literature, our findings do not provide conclusive evidence of a positive or negative association between sleep quality in MCI and conversation to dementia. Studies to investigate associations between sleep and dementia conversion in MCI are subject to inadequate power, subjective measurement of sleep quality, too short follow‐up periods, and inadequate adjustments for confounding. We further assume that self‐reported sleep quality may not be sensitive enough to predict MCI conversion.

### 4.5 Unanswered Questions and Future Research

This analysis failed to detect an effect of sleep quality on dementia in MCI, but showed some evidence for long sleep duration being associated with increased dementia risk. This association is independent of multiple potential confounders but may signal early dementia rather than representing a causal association. It is not clear whether this effect applies to extended sleep alone or the simultaneous presence of sleep disturbances.

Taking into account the many limitations, we classify this study rather an exploratory analysis. Future studies would profit from objective but simple sleep measurements, such as smartphone‐based self‐monitoring devices, objective outcome measurement by experts, and further investigation of potential mechanisms. During past years, research on biomarkers predicting dementia risk gained importance and it has been acknowledged that these play a crucial role in disease progression. On the other hand, the concept of MCI originates from the 1980s and is still based solely on cognitive function. Most likely, patients with MCI are not a homogeneous population but their individual risk for progression to dementia also depends on several non‐modifiable factors (van Maurik et al. 2019). More research is needed to adapt the MCI definition following recent findings emphasizing the pivotal role of genetic characteristics and biomarker‐driven diagnosis.

## 5 Conclusion

In summary, our results support the evidence of an association between sleep duration and dementia conversion in MCI but, considered with previous studies, provide inconclusive evidence about the mechanism behind this association and associations with sleep quality. The study raises important considerations about the limited possibility to analyze the association between sleep quality and dementia conversion in MCI using available population‐based observational studies.

## Author Contributions

**Johannes Schurr**: conceptualization, investigation, writing – original draft, methodology, software, project administration, formal analysis, validation, visualization, data curation. **Hilary Davies‐Kershaw**: writing – review and editing. **Helen Strongman**: writing – review and editing, methodology, supervision.

## Funding

Helen Strongman is funded by the National Institute for Health and Care Research (NIHR) through an Advanced Fellowship (NIHR301730). The views expressed in this publication are those of the author(s) and not necessarily those of the NIHR, NHS, or the UK Department of Health and Social Care.

## Ethics Statement

This secondary data analysis was approved by the Research Ethics Committee of the London School of Hygiene & Tropical Medicine (LSHTM Ethics Reference No. 28555). Ethical approval had been given for each wave of the English Longitudinal Study of Ageing by the National Research Ethics Committee. Details on each ethical approval can be found under https://www.elsa‐project.ac.uk/ethical‐approval.

## Conflicts of Interest

The authors declare no conflicts of interest.

## Data Availability

The data that support the findings of this study are available from the UK Data Service. Restrictions apply to the availability of these data. Safeguarded datasets can be downloaded from https://ukdataservice.ac.uk by registering and accepting UK Data Service's End User License.
